# Recent progress of metal–organic frameworks as sensors in (bio)analytical fields: towards real-world applications

**DOI:** 10.1007/s00216-022-04493-7

**Published:** 2023-01-04

**Authors:** Alessio Zuliani, Noureddine Khiar, Carolina Carrillo-Carrión

**Affiliations:** Asymmetric Synthesis and Nanosystem Group (Art&Fun), Institute for Chemical Research (IIQ), CSIC-University of Seville, 41092 Seville, Spain

**Keywords:** Metal–organic frameworks, Tunable properties, Analytical tools, Sensors, Contaminants, Bioanalysis

## Abstract

The deployment of metal–organic frameworks (MOFs) in a plethora of analytical and bioanalytical applications is a growing research area. Their unique properties such as high but tunable porosity, well-defined channels or pores, and ease of post-synthetic modification to incorporate additional functional units make them ideal candidates for sensing applications. This is possible because the interaction of analytes with a MOF often results in a change in its structure, eventually leading to a modification of the intrinsic physicochemical properties of the MOF which is then transduced into a measurable signal. The high porosity allows for the adsorption of analytes very efficiently, while the tunable pore sizes/nature and/or installation of specific recognition groups allow modulating the affinity towards different classes of compounds, which in turn lead to good sensor sensitivity and selectivity, respectively. Some figures are given to illustrate the potential of MOF-based sensors in the most relevant application fields, and future challenges and opportunities to their possible translation from academia (i.e., laboratory testing of MOF sensing properties) to industry (i.e., real-world analytical sensor devices) are critically discussed.

**Graphical abstract:**

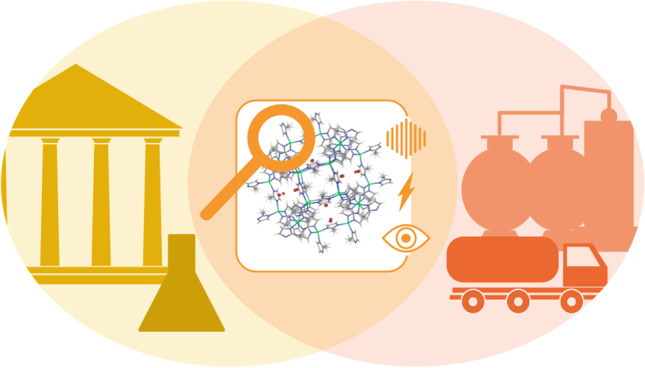

## Introduction

During the last years, the R&D in analytical chemistry has made great strides in designing sensors, i.e., analytic devices responding to the presence (qualitative sensors) and/or concentration (quantitative sensors) of a specific target analyte, a family of compounds, or several analytes simultaneously (multiplexed sensors), for central applications in industrial process management, chemical risk detection, food quality control, environmental analysis, and medical diagnosis. Although the well-known 3 “S” rule, i.e., sensitivity (the slope of the calibration curve), selectivity (the ability to discriminate a target analyte), and stability/reusability (changes in accuracy in function of time/cycles of use), defines the crucial characteristics to pursue when designing a sensor, the most recent trends in the field are aimed at preparing analytical sensors also featuring the following [1]:

(i) Fast response. This is particularly important when a quick decision needs to be made for health issues, economic reasons, or environmental remediation. For example, rapid analysis is necessary in contaminated sites, to instantly provide data for the assessment of the remediation activities. Rapid analysis is also needed in pandemic situations, to monitor the spread of the disease, such as in the case of the COVID-19 pandemic.
(ii) Portability. Portable sensors present a clear advantage, for example, in fighting the disposals of toxic reagents in sites difficultly monitored or in tiny environments. Another big area that is enormously benefiting from the rapid evolution of miniaturized sensing systems is the medical field, as evidenced by the increasing use of wearable sensing devices.
(iii) Easy and low-cost methods. The easiness and simplicity in the preparation of sensing materials (e.g., one step synthesis, low-temperature, and low-pressure conditions) are characteristics that facilitate further large-scale production, also contributing to cutting costs. Low-cost sensors are especially advantageous in low- and middle-income countries to increase their quality of life, as the recent COVID-19 pandemic has made even more visible.
(iv) Sustainable features. In the context of analytical sensors, the concept of sustainability means developing both the sensing materials and the entire devices through environmentally friendly procedures (e.g., reducing waste production or lowering the energetical consumption of the synthetic procedures), by using sustainable sources of materials or green methods for the preparation of the sensing materials, or by developing recyclable sensors (i.e., with a long lifespan, and with possible reuse of their individual components).

**Fig. 1:**
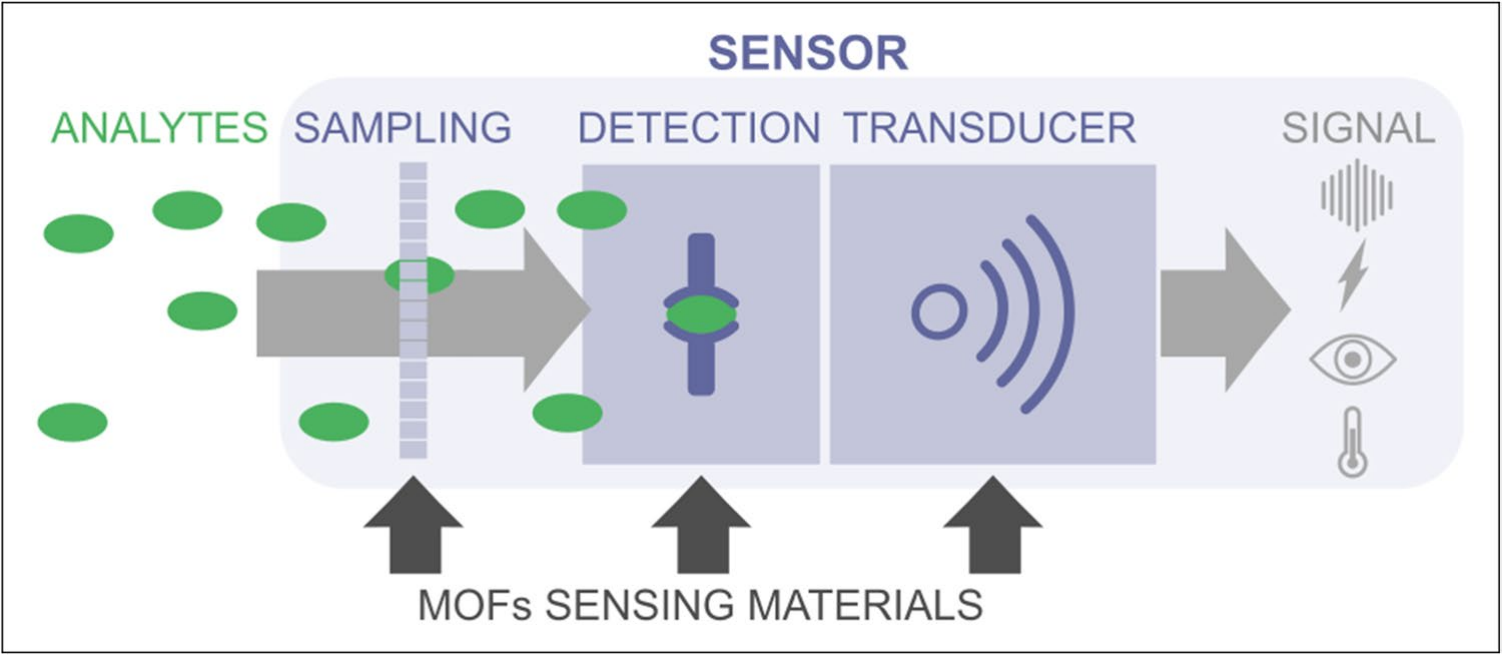
Scheme of the main parts of a sensor (sampling, recognition, and transducer), showing that MOFs can play a functional role in the different parts, either as a sorbent material, as a sensing/recognition material, and/or as a transducing element

The achievement of all these desired characteristics implies multidisciplinary strategies focused on innovating in the different components of a sensor, either in the sampling unit (for the capture of the analyte, and separation/filtering from the matrix interferents), in the recognition phase (where the interaction of the analyte with the sensing element takes place and undergo a chemical change), or in the transducer (to convert the chemical change into a measurable physical signal that can be optical, electrical, thermal, mass or acoustic); see Fig. 1.

More than 25 years of research on reticular chemistry, i.e., the chemistry of systems formed by molecular building blocks regularly interconnected to form solid-state materials in a designed and rational manner, have led to the preparation of a huge number of materials for multiple applications, including (bio)chemical sensing materials [2]. Among the reticular materials, metal–organic frameworks (MOFs) are on a more mature phase of research, including significant advances in MOF-based sensors. A MOF is basically the extended crystalline structure of metal-containing inorganic building units (metal nodes) connected to multidentate organic building units (linkers) via coordination bonding. The units of metal nodes, organic linkers, and sequentially structural motifs yield an essentially infinite number of possible combinations to form a MOF [3]. Indeed, more than 100,000 different structures of MOFs have been reported up to date, offering more possibilities of developing sensing materials than the ca. 600 structures reported for COFs [4].

The main advantages of using MOFs for designing sensing materials rise from their unique and highly tunable physicochemical/structural (and hence functional) characteristics [5], including their regular porosity and tunable pore size, multivariate structures with multiple metals (either mono-, bi-, or tri-metallic systems) and/or organic linkers, to even employing conformationally flexible linkers and/or geometrically versatile inorganic building units. In addition, MOFs can be functionalized (both the external surface and internal pore space) through post-synthetic modifications (PSM) to install new functionalities. More recently, the engineering of partially aperiodic frameworks, to purposefully introduce building-block vacancies and heterogeneities, is attracting much interest to fully exploit the potential of MOF chemistry. All these features can (at least theoretically) address the need of developing new sensing materials featuring high sensitivity, high selectivity, quick response time, enhanced stability, and reusability. If at first glance sensitivity appears to be more related to modification of the surface chemistry of MOFs, for example, by specific (bio)chemical interactions of the analyte with functional groups or opens metal sites (OMS) [6], and selectivity seems merely determined by pore and aperture sizes, for example, by size exclusion of larger molecules, the simultaneous combination of both surface chemistry and pore dimensions can synergistically enhance sensitivity and selectivity [7], such as in the case of specific interactions of a selected analyte with the functional groups placed in the internal surface of the MOFs or intentionally created vacancy defects. Concerning the response time, this depends mainly on the rate of diffusion of the analyte to the interacting site, thus connected to the MOF particle size and pore dimensions of the MOFs [8]. Bearing this in mind, nanosized MOFs, which have higher surface-to-volume ratios in comparison with micrometer-sized MOFs, show faster diffusion rates and therefore faster response to analytes, being usually preferred to ensure rapid analyte uptake and equilibration with higher sensitivity. Nanosized MOFs are also selected for sensing applications in the biomedical field, since smaller particles are more efficiently internalized in cells. On the other hand, the long-term stability in operation/detection conditions and, therefore, the reusability of MOFs, is a critical issue that still requires much improvement. Many studies have focused on improving the kinetic and/or thermodynamic stability of MOFs by introducing hydrophobic substituents, by changing the MOF constituents to increase the strength of metal–linker bonds [9], or by selecting inner clusters (e.g., Zr_6_-cluster) with the ability of reversibly rearrange upon removal or addition of μ_3_-OH groups, without any changes in the connecting carboxylates [10].

Nevertheless, despite the tremendous progress that has been made to date in the design of MOF-based sensing materials, the development of sensors with industrial relevance and application in real applications still requires intense research to solve the main current limitations. Firstly, the selectivity is normally poor. Many MOF-sensing approaches entail a loss of signal (i.e., fluorescence or phosphorescence intensity) in response to the interaction/adsorption of the analyte; these “turn-off” sensors can also be affected by other interference which also results in signal loss, ultimately resulting in increased LOD. Moreover, some MOFs still suffer from the drawback of relatively poor stability under working conditions.

Besides, some limitations related to the production of MOFs at an industrial level should be considered. Currently, the synthesis of many MOFs is expensive, mainly due to the cost of organic linkers. Moreover, the processability of MOFs is hampered by the powdery nature of these materials, considering also the health issues related with the processing of nanoparticles, since many MOFs are in the nanoscale dimension. Unfortunately, there is very limited data (especially in real-life conditions) regarding the health risks related to nanomaterials [11]. Additionally, the synthesis of MOFs under the principles of green and sustainable chemistry is scarcely considered today, and only a few studies have carried out life cycle assessments of MOFs [12–14].

Therefore, the development of MOF-based sensors with future application potential in real scenarios remains an open challenge, and it requires more research efforts focused on new MOF-based sensing materials as well as on new efficient, sustainable, and scalable synthetic strategies.

Several excellent reviews related to the potential of MOFs for different (bio)sensing applications have been published over the last couple of years, but each is approached from a different perspective. While some reviews discuss very specific types of MOFs or composites (such as carbon dots@MOFs [15] or lanthanide MOFs [16]), others are focused on specific sensor types (e.g., electrochemical [17–20] or luminescent [21]), specific analytes (e.g., food contaminants [22], gases [23, 24], neurotransmitters [25], biomarkers for cancer [26]), or specific applications fields (e.g., biomedical, food) [27, 28]. In contrast, this review presents an overview of the potential of MOFs for real-world sensing applications, highlighting recent trends in the most relevant (bio)analytical fields based on figures and emphasizing current limitations and challenges that must be faced. Therefore, the review is organized into three sections. First, the key properties of MOFs to be used as sensing materials are introduced. Second, a section summarizes the use of MOFs as sensing materials in different application fields, including food control, environmental analysis, and biomedical purposes, reporting some representative analytes. In a final section, the challenges and prospects of the nearest future of MOF sensors are critically discussed, and some patents are also reported to illustrate the growing interest of MOFs as sensors in the field of industry.

## Key features of MOFs for sensing

As previously mentioned, the key characteristics of MOF-based sensors are sensitivity, selectivity, response time, stability, and reusability, as well as incorporating appropriate signal transduction capabilities (e.g., optical, electrical/electrochemical, photoelectrochemical, mechanical, thermal, mass, magnetic, acoustic). Moreover, in some sensing schemes and with the future perspective of developing portable sensors, the construction of analytical sensing devices involves the fabrication of films of MOFs (which are normally primarily obtained as powders) either through the deposition or coating of substrates with MOF crystals. Bearing all this in mind, it seems clear that the preparation of the MOF for each specific sensing application must follow a “design-for-purpose” approach to ultimately achieve the desired sensor performances.

### Selectivity and sensitivity

The two main strategies to enhance the selectivity of MOF-sensing materials are size exclusion (i.e., molecular sieving by pores) and physical–chemical interactions. The MOF porosity can be varied through composition, that is judiciously selecting the metal ions and organic linkers (e.g., node and linker sizes and geometries, linker appendages and their directional orientation), which allows not only to change the pore and aperture sizes but also its hydrophilic-hydrophobic character to increase the affinity towards the target analyte. While many MOFs are microporous materials (having pores of  < 2 nm), depending on the molecular dimensions of the target analytes MOFs with large pores, either mesopores (2–50 nm) or even macropores (> 50 nm) are required. Pore sizes in MOFs can be enlarged by increasing the separation between metal nodes using long organic linkers. However, this method fails with very long linkers since interpenetrated networks with small pore sizes are usually formed. Strategies to overcome this effect include the use of sterically hindered linkers [29], the use of sacrificial templates [30], or the replacement of node-coordinated molecules (e.g., solvents) with other ligands [31]. Concerning the role of physical–chemical interactions as strategy to modulate the selectivity, as well as to increase the sensitivity, the incorporation of functional groups (e.g., amines, carboxylic acids, hydroxyls) within the framework is commonly used to favor the binding with the target analyte through hydrogen bonding, electrostatic interactions, electron donor/acceptor interactions, or covalent bonds formation. These groups can be incorporated in the MOFs during the synthesis (by using organic linker containing already those groups), or through post-synthetic modification (PSM) approaches by modifications on the linkers [32] or on open metal sites (OMS, known also as coordinatively unsaturated sites (CUS) or occasionally also as open coordination sites (OCS) [6]) [33]. For example, TMU-60 was synthesized using ligands with amino groups to form electron-rich pores capable of detecting different (electron-poor) metal ions such as Pb^2+^, Hg^2+^, Cu^2+^, Cd^2+^, Cr^2+^, Ni^2+^, and Cr^3^ [34]; while Mg-MOF-74 films with open metal sites were post-synthetically modified with ethylenediamine and used as sensing materials for the detection of CO_2_ and benzene [35]. More complex functional moieties can be also incorporated, such as biological recognition elements (i.e., antibodies, enzymes, DNA strands), forming the so-called biosensors that are capable fr endowing the MOFs with a higher level of selectivity or even specificity towards some analytes.

When it comes to sensitivity, although it depends to some extent on the method of signal transduction, the strength of the analyte binding to the MOF will have a strong influence; thus, stronger binding will lead to lower detection limits. The strength of the analyte-MOF can be adjusted by also playing with the size of the pores, the hydrophilic-hydrophilic nature of the pores, the presence of specific functional organic groups, or the functionalization with biomolecules. For example, small pores will adsorb gas or vapor analytes more strongly than large ones, resulting in an enhanced sensitivity [36], such as in the case of the detection of H_2_S with ZIF-8 [37]. Furthermore, ultra-high surface areas and overall dimension of MOFs can determine better sensing performances, especially in the case of nanosized MOFs due to their higher surface-to-volume ratio, which translates into higher sensitivity as well as faster response because of the shorter diffusion paths. MIL-96(Al) nanoparticles (ca. 200 nm diameter), for example, showed high sensitivity towards water detection [38]. More recently, the creation of defects within the MOF structure (i.e., open metal sites) has also been proposed as a strategy to promote preferred analyte binding for selective detection. Sensitivity can be also enhanced by signal amplification approaches, exploiting specific guest molecules or inorganic metal particles grafted, supported, or encapsulated on/in the MOFs, such as DNA, metal nanoparticles, carbon dots, and carbon nanotubes [39, 40]. For example, the modification of Cu-MOFs with DNA chains resulted in enhanced sensitivity towards Pb^2+^ [40] or miRNA detection [41].

As highlighted in Table 1**,** the different approaches for boosting the selectivity and the sensitivity present divergent advantages and disadvantages, which should be taken into account in the design of the sensors depending on the final application; for example, highly selective but expensive sensors may be justified in the specific case of biomedical use.

**Table 1:**
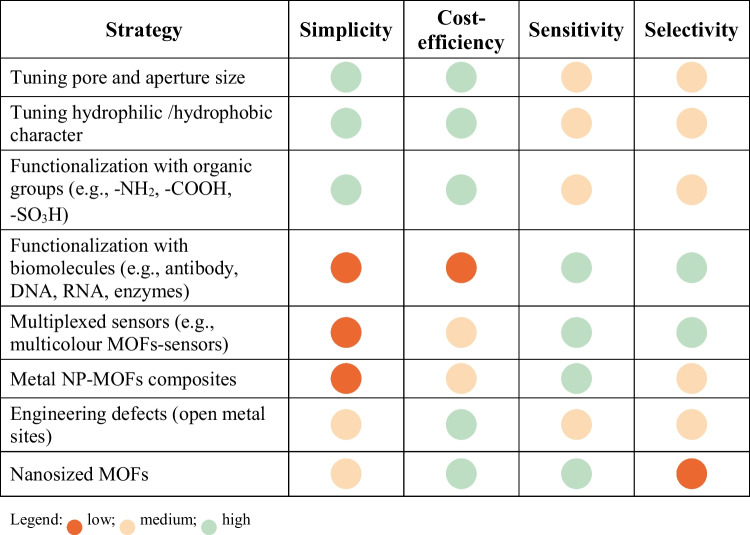
Advantages and disadvantages of the main strategies for enhancing selectivity and sensitivity

### Stability

The stability of MOFs derives from different factors such as the type, geometries, and properties of the linkers and of the metal nodes, the strength of the coordination bonds, and the MOF topology, as well as the particle sizes of the MOFs (nanoMOFs are normally less stable than microsized MOFs or bulk MOFs). Among all, the strength of the M-linker has been pointed out as the most determining factor of the stability of MOFs, and more importantly, it can be designed to be strong enough not to break under specific sensing conditions. Theoretically, Pearson’s hard/soft acid/base principle (HSAB) can be used to predict and assume the stability of M-linker. Thus, in order to prepare highly stable MOFs, metal ions with high valence states (e.g., Ti(IV), Zr(IV), Al(III), Fe(III) and Cr(III)), considered as hard acids, should interact with hard bases ligands (e.g., carboxylate ligands), while soft bases ligands (e.g., imidazolate, pyrazolate, triazolate) should interact with soft metal ions (e.g., Zn(II), Cu(II), Mn(II)) [42]. For example, Zr-based MOFs synthesized using tetracarboxylate ligands possessed such high stability and durability that were employed as stationary phase in HPLC [43]. In addition, ligand rigidification has been demonstrated to be an efficient approach for enhancing the stability of MOFs. For instance, ligands with high rigidity, such as tetratopic ligands in Zr-based MOFs, have a high energy barrier and tend to keep their conformation, stabilizing the structure of MOFs [44]. Improvements on the MOF stability can also be achieved by PSM. This is the case of a highly stable PCN-777 luminescent sensor for protein detection prepared by the post-synthetic addition of ligands enriched with carboxylic groups, forming strong bonds between Zr^4+^ and -COO^−^ [45].

### Rapid response time and reusability

A fast response after interaction with the target analyte and the possibility of using the MOFs for different cycles depend mainly on the thermodynamics and kinetics of adsorption. More in detail, the rate of diffusion of the analytes to the interacting part of the MOFs (either within the internal pores or the external surface) is determined mainly by (i) intrinsic properties of the analyte, e.g., molecular dimension, flexibility, polarity; (ii) properties of the MOF such as the size, geometry and nature of the pores, MOF particle size (since diffusion times increase as the square of diffusion distance), MOF morphology (e.g., 2D nanosheets MOFs lead generally to superior sensing properties because of enhanced diffusion and accessibility of analytes to the recognition sites), and the “breathing” effect of the MOF; and (iii) the medium (solvent) when the sensor is used in solution, or the thickness of the MOFs films when used as a layer onto a solid support. For example, a thin film of MFU-4-based sensor for deuterium detection showed a fast response time of only a few milliseconds [46]. Similarly, NTU-9 nanosheets showed fast response through Fe^3+^ detection thanks to high dispersive nature and highly accessible active sites [47]. In addition, the presence of coordinated solvent molecules or other guest molecules (e.g., modulators used in the synthesis) inside the pores of the MOF can slow the diffusion of the analytes. This is why the activation of MOFs for the efficient removal of such potential guest molecules, which is usually done by heat and/or vacuum treatment, is so important. On the other hand, since most of the analytes are physisorbed, the reusability of the MOFs can be achieved by vacuum treatment aided by heating, such as in the case of KAUST-8 used as sensing material for SO_2_ and regenerated by heating at 105 °C in vacuum [48]. Reusability is clearly not considered for MOFs sensing probes designed for single use (i.e., when the materials go through irreversible reactions).

### Signal transduction strategy

Although, in principle, any MOF property that changes upon interaction with the analyte could be measured as a sensing signal, most MOF sensors reported to date are based on either optical transduction schemes (i.e., luminescent, colorimetric, or plasmonic) or electrochemical. Note that the incorporation of nanoparticles (NPs) within the framework, for example, luminescent quantum dots or plasmonic Au NPs, is another approach to form MOF-based composites having multifunctionalities for improved signal transduction mechanisms.

(i) Luminescent sensors. In luminescence sensors, the MOF works as both recognition material and transducer; thus, the photoluminescent properties of the MOFs must be finely tuned in order to have detectable signals (i.e., not too quenched). The photoluminescence of MOFs can arise from (a) the luminescence properties of organic linkers (normally extended π-conjugation systems with rigid structures), such as in MOFs containing pyrene, anthracene, and similar compounds as building units; (b) metal-based emissions, such as in lanthanide-based MOFs; (c) metal–to–ligand charge transfer (MLCT), such as those occurring in Cu(I) or Ag(I) based MOFs; (d) ligand–to–metal charge transfer (LMCT), for example, in Zn(II)/Cd(II) and carboxylate ligands based MOFs; (e) ligand–to–ligand charge transfer (LLCT), known as antennae effect, which involves the indirect excitation of the metal such as in the case of MOFs with absorbing ligands (e.g., π- and σ-bonded antenna ligands) and emitting lanthanide ions; or (f) fluorophores loaded as guest molecules in the MOF pores, such as guest-centered emission and guest-sensitization. From the perspective of the metal-type MOF, the vast majority of luminescent MOFs can be classified into two groups: lanthanide-based MOFs (Ln-MOFs) and transition metal-based MOFs [49] Ln-MOFs are very useful for sensing purposes due to their unique luminescent properties such as long lifetime, sharp emissions, and high quantum yields in the visible and near-infrared (NIR) regions. Notably, the possibility of working in the NIR region makes Ln-MOFs compatible for in vitro (cells) and in vivo (animals) sensing applications, avoiding the typical cell and tissue autofluorescence in the blue-green region. Besides, binary and ternary co-doped Ln-MOFs can be easily prepared, and these mixed Ln-MOFs can generate simultaneous emission of different Ln ions using one single excitation wavelength, resulting thus in MOF sensors with improved sensitivity and selectivity [50]. Among the luminescent transition metal-based MOFs, Zn- and Cd-MOFs are the most commonly reported since d^10^ metal ions have several coordination numbers and geometries and exhibit luminescent properties when bound to functional ligands. In contrast to Ln ions, transition metals generally do not have intrinsic luminescence, but they modulate the MOF emission by participating in LMCT or MLCT processes. Whereas LMCT is often reported in Zn- and Cd-MOFs, MLCT is more frequently found in Cu- and Ag-MOFs [51].
(ii) Colorimetric sensors. These sensors have attracted increasing attention due to their simple and rapid signal readout with the naked eye or using smartphones, which allows for in situ sensing applications [52]. In a colorimetric sensor, an optical shift in the visible absorption band of the sensor is produced after adsorption/interaction with a specific compound due to intermolecular interactions. MOFs with tunable colorimetric responses can be obtained through careful design, either using transition metal ions or chromophore ligands. upon the introduction of analytes. Alternatively, chromophores can be loaded into the internal pores or attached to the MOF surface. Compared with individual metal ions or chromophores as colorimetric probes, the use of colorimetric MOFs has several advantages, such as superior stability due to the protection provided by the framework to the chromogenic reaction, and enhancing detection sensitivity and selectivity thanks to the intrinsic porosity, open metal sites, and Lewis acid/base sites, which can be adjusted on purpose.
(iii) Electrochemical sensors. These sensors offer the possibility of detecting in a simple and fast way analytes that can be easily oxidized or reduced, by means of changes in the measured current, electric potential, or other electrical signals. Since electrochemical reactions can only occur on the electrode surface, the deposition or immobilization of the MOFs on the electrode surface is required. The preparation of MOFs with electrocatalytic activity involves the introduction of the redox-active sites in the metal nodes or organic ligands, such as the incorporation of active metal sites with nitrogen-containing ligands (e.g., porphyrin- and bipyridine-based ligands) or the functionalization of ligands with electron-donating or electron-withdrawing groups. In addition, the excellent electrocatalytic performance reported for some MOF-based structures arises from their high porosity (which result in rapid mass transport during electrochemical reaction, improved adsorption capacity, and reduction of the activation energy of the intermediates), and the selective interaction of the analyte with the catalytic sites in the MOF (which improves the selectivity of the electrochemical response). However, due to the fact that the majority of MOFs are insulating, the MOFs are subjected to high-temperature annealing to transform non-conductive or low-conductive MOFs to conductive materials for achieving a good electrocatalytic response; the MOFs are thus used as sacrificial templates since the unique structures and intrinsic active sites in MOFs are destroyed during the thermal treatment. Three main strategies are proposed to improve the electrocatalytic activities of MOFs in their pristine form [8]: preparing conductive or electrochemically active MOFs, MOFs supported on conductive substrates, and MOFs hybridized with active materials. It has also been shown that nanoMOFs and ultrathin 2D MOF nanosheets present a significantly higher electrochemical activity, which is related to the presence of more active sites exposed or easily accessible, together with the improved diffusion of the analyte to the catalytic sites.

It is worth noting that in electrochemical sensing, MOFs are typically deposited on glassy carbon electrodes (GCEs) or other substrates to promote integration of MOFs with portable electrochemical devices; therefore, the development of efficient methods for the fabrication of these MOF-based films is very importance in these sensing schemes.

### Fabrication of MOF films

The integration of the MOFs onto surfaces and devices is required for some MOF-based sensors (i.e., solid-state sensing applications), in which the signal transduction method requires a physical interface between the MOF and the device support. This is, for example, the case in electrochemical sensing. It should be noted that the properties of the MOF films such as the crystal size and thickness affect their electron-transfer kinetics and adsorption ability, and thus achieving a precise control of all these parameters is crucial to achieve desired film properties such as variable thickness ranging from nanometers to a few micrometers and low roughness.

MOFs films are usually prepared through two main strategies [53–56]: (i) by direct synthesis of MOFs on the support, via dip coating, film coating, interfacial synthesis, evaporation method, electrochemical approaches, etc., or (ii) by deposition of already-synthesized MOFs by simple evaporation onto the support, self-assembly of MOFs to form monolayer further deposited on a support, or by incorporation of MOFs into gel or polymeric matrices. In the first case, the film formation can be accomplished by reacting the linkers and the metal precursor directly on the surface, which is often previously modified with a self-assembled monolayer or with small MOF crystals to facilitate the nucleation process. The presence of functional groups on the surface can also favor the nucleation of MOFs to a crystallographic direction or lead to coordination geometries distinct to those normally obtained by standard synthesis. This is the case of a recently reported work in which a film of Zr-based MOF was prepared by vapor-assisted conversion and oriented along the [111] crystal axis, thanks to the modulation of the surface modifiers, the droplet volume, and the reaction time [57]. The other approach for the preparation of MOF films consists in preparing MOF particles (preferably small particles) and sequentially depositing them on a surface by different techniques. For example, NH_2_-MIL-88B was deposited by spin coating on a silicon wafer for the preparation of colorimetric sensors for various volatile organic compounds (VOCs) [58].

## Overview of the applications of MOFs as sensors in different fields

**Fig. 2:**
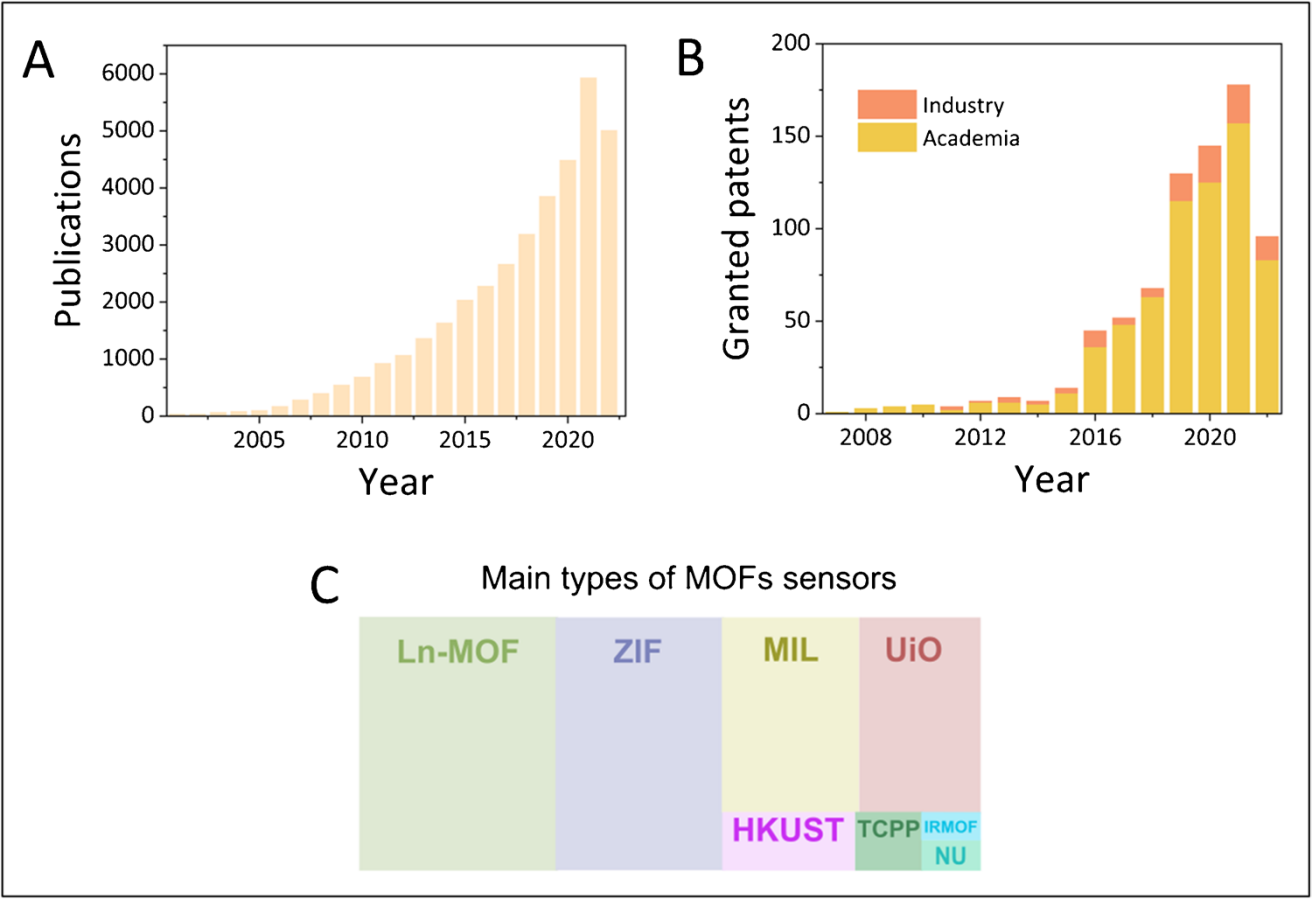
**A** Publications (2007–2022) and **B** patents (2008–2022, early publication date) per year from WOS searches with the topic “metal–organic framework (or MOF) sensor, detection, quantification, analysis.” Taken on the 13th of October 2022. **C** Tree map of the main families of MOFs used in sensing applications from WOS searches taken on the 7th of December 2022. ZIF, zeolitic imidazolate framework; MIL, Materials Institute Lavoisier; UiO, University of Oslo; HKUST, Hong Kong University of Science and Technology; TCPP, tetrakis(4-carboxyphenyl)porphyrin; IRMOF, isoreticular metal–organic framework; NU, Northwestern University

**Fig. 3:**
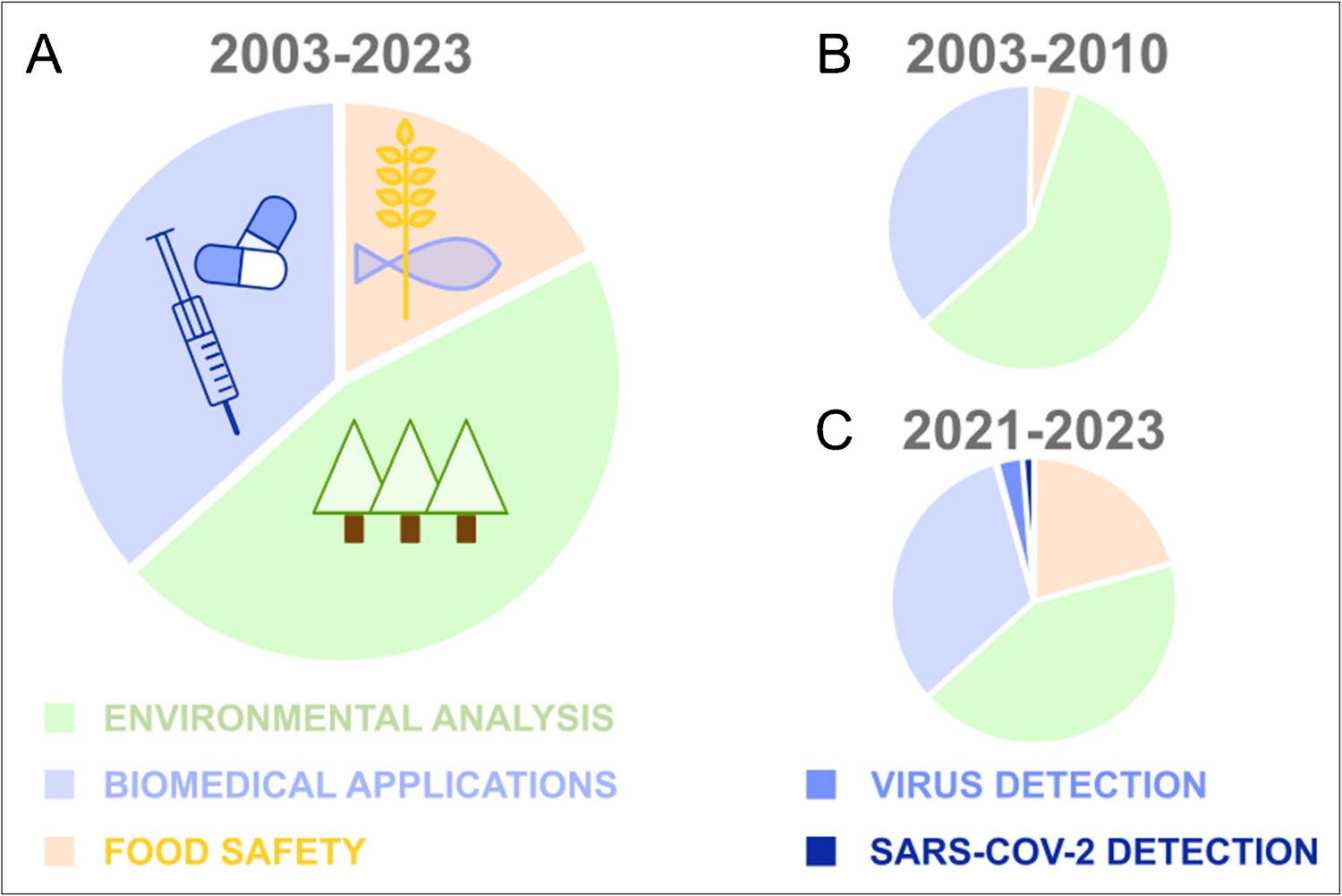
Segments of publications in the three main research areas (i.e., environmental analysis, food control, and biomedical applications) from WOS searches taken on the 14th of October 2022

The research on MOF-based sensors has generated numerous innovative outcomes, as evidenced by the exponential increase in publications and patents during the last years in this area (Fig. 2A, B). Considering the types of MOFs reported in the publications, it is observed that they belong mainly to the families of lanthanide MOFs (Ln-MOF), ZIF, MIL, UiO, and HKUST, and to a lesser extent to TCPP-based MOFs, IRMOF and NU (Fig. 2C). Regarding the fields of application of the publications during the last two decades, it is observed that they mainly belong to environmental, food control, and biomedical areas, with significantly distinct proportions of each one (Fig. 3A). If in the early 2000s the focus on environmental analysis was more consistent (green segment of the pie graph in Fig. 3B), during the last two years, the research on sensors for food control has gained more attention, while the percentage of publications related to the biomedical area (ca. one-third of the annual publications) remained fairly the same since 2003. Notably, in the last 2 years, there has been a growth of interest in virus diagnosis, with a special focus on the subcategory of the SARS-CoV-2 diagnosis (blue and dark blue segments in Fig. 3C, respectively).

On the other hand, it must be highlighted that most of the granted patents belong to the academic sector, and only a few of them to the industry (Fig. 2B), which underlines that the shifting to real-world applications remains an open challenge. Nevertheless, the trend of the last period indicates that private companies are gradually paying more attention to intellectual property rights, reflecting the growing interest in the use of MOFs in industrial applications. This tendency is expected to rapidly grow whenever new technologies and processes for preparing MOF sensors will overpass current limits for large-scale production, as further discussed in the final section.

Regardless of the final application, the success of the utilization of MOF-based sensors depends strongly on the capacity of delivering valid analysis compared to traditional analytical techniques. Up to date, those include classical techniques such as high-performance liquid chromatography–mass spectrometry (HPLC–MS), inductively coupled plasma mass spectrometry (ICP-MS), and gas chromatography–mass spectrometry (GC–MS) [59]. Other analyses specific for biomarkers and pathogens include polymerase chain reaction (PCR) [60], enzyme-linked immunosorbent assay (ELISA), lateral flow immunoassay, flow-through immunoassay, or surface plasma resonance (SPR). Capillary electrophoresis–mass spectrometry (CE-MS) and NMR are also widely used considering the complexity of many biomedical analytes [61]. Volatile organic compounds (VOCs) are instead analyzed by the same GC and LC techniques often coupled with spectroscopy (MS), time of flight (TOF), and thermal desorption (TD), or by selected-ion flow-tube mass spectrometry (SIFT-MS) and proton-transfer-reaction mass spectrometry (PTR-MS). Most of these techniques show high selectivity and low detection limit but are often time-consuming and expensive. In addition, they frequently require complex pretreatment or need to be run by expert users. Lastly, they cannot perform real-time measurements. Thus, the development of highly sensitive yet simple MOF-based sensors offers valuable alternatives to current techniques. The most relevant types of analytes to be detected with the newest MOF-based sensors for food control, biomedical diagnosis, and environmental monitoring are here summarized, pointing out the importance of each sector for the community.

### Food control

According to the World Health Organization (WHO), it is estimated that every year over 600 million people fall ill after eating contaminated food. Of those, more than 400,000 individuals suffer severe consequences leading to death [62]. Besides, food contamination causes 110 US$ billion lost yearly [62] due to waste generation in production processes and due to medical expenses mainly in low- and middle-income countries. Food contamination can be caused by many harmful bacteria, viruses, parasites, chemicals, and heavy metals which are responsible for more than 200 diseases, ranging from fever to cancer. Food safety is achieved by high-quality standard operating procedures (SOPs), and by the analysis of food at each phase of the food supply chain. Indeed, major food hazards can enter the food at any time during harvesting, processing, transporting, and storing. Thus, the use of fast, on-site, and cost-effective analytical devices is crucial. In recent years, MOFs have been proposed as alternative materials for the design of sensing elements for food safety analyses especially thanks to enhanced photo/thermal stability and high selectivity.

Among the different types of food contaminants, MOF-based sensors have been recently used to detect [22]: (i) pesticides, such as organophosphorus pesticides and organochlorine pesticides; (ii) antibiotics, including tetracyclines, cephalexin, and chloramphenicol; (iii) pathogens such as *Staphylococcus aureus* and *Escherichia Coli*; (iv) natural toxins which can be highly carcinogenic, teratogenic and mutagenic, including ochratoxin A and aflatoxin B1; (v) heavy metals; (vi) persistent organic pollutants (POPs) like polychlorinated benzenes; and (vii) biogenic amines such as of putrescine (PUT) and cadaverine (CAD).

**Fig. 4:**
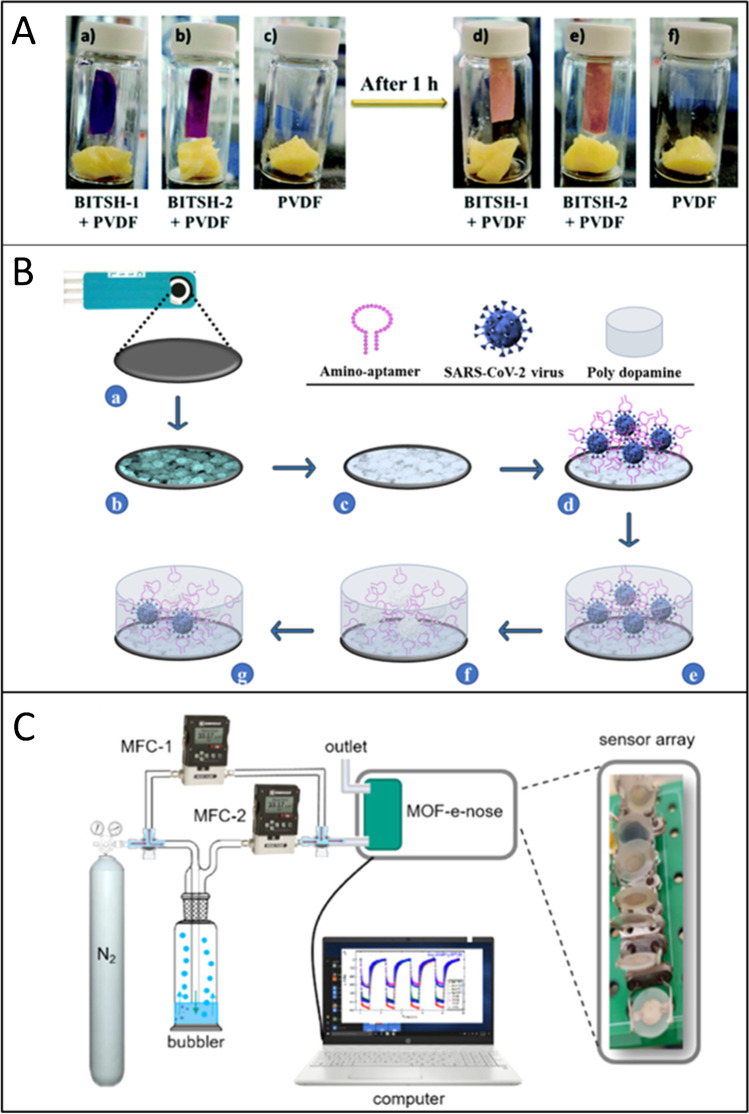
**A** Visual detection of biogenic amines from cheese using a PVDF polymeric membrane modified with Co-based MOFs (BITSH-1 or BITSH-2) as colorimetric sensors. Used with permission of RSC, from [63]; permission conveyed through Copyright Clearance Center, Inc. **B** Schematic illustration of the fabrication process of the MOF-based aptasensor for the detection of SARS-CoV-2 virus. Reprinted (adapted) by permission form Springer [79], copyright 2022. **C** Scheme of the electronic nose setup, composed of an array of QCM sensors coated with MOF films of six different structures. Vapor concentration is controlled by the flow rates through the mass flow controller and sensor array is located in a gas flow chamber. Reprinted (adapted) with permission from [90]. Copyright 2022 American Chemical Society

To simply show a recent example in this area, two new Co-based MOFs, BITSH-1 and BITSH-2 (Birla Institute of Technology and Science, Hyderabad), were reported for the sensing of PUT and CAD, as key markers to monitor food spoilage in protein-rich foods [63]. The UV adsorption spectra of these MOFs showed strong absorption and emission bands at 278–280 nm and 332–333 nm, respectively. The fluorescence intensity of the MOFs was quenched by small addition of PUT and CAD and was ascribed to photoinduced electron transfer between the biogenic amines and the MOFs. The LOD values achieved were lower than those obtained with other MOFs thanks to a higher HOMO level (∼ − 5.91 eV) of the BITSH-1 and BITSH-2, which facilitated the electron transfer from the HOMO of PUT and CAD to the HOMO of the MOF. Importantly, the formation of a polymeric membrane modified with these MOFs resulted in a simple visual sensor, revealing changes of color upon exposure to real food samples (Fig. 4A).

### Biomedical area

A global increase in the quality of life requires excellent healthcare systems. Although the improvement of medical care has been already pointed out in 2015 as the key to address multiple targets of the UN’s SDGs “Ensure healthy lives and promote well-being for all at all ages,” the COVID-19 pandemic has further highlighted this need [64]. In this context, analytical chemistry plays a crucial role in developing tools for the detection of diseases and in providing analytical data for the optimization of medical treatments. MOFs sensing materials have emerged for biomedical applications principally due to their low cytotoxicity (which is critical for eventual in vivo applications), intrinsic biodegradability, biological affinity, and the possibility of integrating biomolecules in the MOF structures (i.e., to prepare biosensors). Recently developed MOF sensing materials have been used for the detection of (i) pharmaceutical drugs [65]; (ii) biomarkers for non-communicable diseases, such as cardiac troponin (myocardial injuries) [66], microRNA (different disease states) [67], or VOCs (in the breath of patients and related to different diseases) [68]; (iii) specific biomarkers for communicable diseases (e.g., tuberculosis, malaria, hepatitis, HIV/AIDS), such as MPT64 and ESAT-6 proteins (biomarkers secreted by *Mycobacterium tuberculosis*) [69, 70], or dipicolinic acid (biomarker for anthrax) [71]; (iv) pathogens (virus, bacterium, or fungus), such as *Pseudomonas Aeruginosa* [72], hepatitis-C [73], human immunodeficiency virus type 1 (HIV-1) [74], and Ebola [75].

Within the biomedical area, a special focus should be given to the sensors for the detection of the SARS-CoV-2 virus. Indeed, the COVID-19 pandemic has generated unfortunately well-known socio-economic impacts (for example, the WHO has estimated that the total deaths directly or indirectly related to the pandemic during 2020 and 2021 were between 13.3. million and 16.6 million). Thus, researchers around the world have been intensively investigating different strategies for the efficient, selective, and rapid detection of SARS-CoV-2, both considering the biomarkers, or the pathogen. Currently, the most widely used technique in clinical laboratories is the reverse transcriptase–polymerase chain reaction (RT-PCR). However, this technique has some important limitations, including false-negative rate of up to 37%, long analysis time (4 h on average), and the uncomfortable sampling procedure (especially for infants). Some MOF-based sensors have been developed as valid alternatives for the specific detection of COVID-19 [76], such as a highly sensitive, relatively low-cost, and fast-response (only 5 min) sensor based on UiO-66 to determine the SARS-CoV-2 spike protein [77], or a fluorescent MOF-5/CoNi_2_S_4_ sensor decorated with porphyrin for efficient detection of recombinant SARS-CoV-2 spike antigen [78].

Here again to show just one example of MOF-based sensor relevant to this area (Fig. 4B), a MOF-based aptasensor was developed using a screen-printed carbon electrode (SPCE) modified with a Ni-MOFs, specifically Ni_3_(BTC)_2_ functionalized with the aptamer [SARS-CoV-2 virus], and it was used successfully for the detection of intact form of the SARS-CoV-2 virus [79]. The analytical performance of the sensor was evaluated by means of electrochemical impedance spectroscopy (EIS) using [Fe(CN)_6_]^3−/4−^ as redox probe. The results showed an increase of the charge transfer resistance upon incubation with increasing concentrations of the virus, due to the formation of more SARS-CoV-2 virus–aptamer complexes. The selectivity of the sensor was investigated by incubation with several potential types of possible interferers such as MERS-CoV, influenza A H1N1, and influenza A H3N2, observing no significant response was observed for off-target species. Importantly, the analysis of real samples of sick and healthy individuals showed a 100% sensitivity with 100% specificity. The sensor was also usable after 14 days and stored at 4 °C, demonstrating good long-term stability.

### Environmental monitoring

The enormous industrial and social development of the last century and even more in recent decades has intensely increased environmental pollution, generating severe problems for the ecosystem and society [80–82]. Indeed, environmental pollution affects deforestation, acid rain, greenhouse effect, etc., and generates health problems such as cancer, asthma, inflammation, and many other diseases. For example, only in 2016, 7 million deaths resulted from indoor and outdoor pollution (note that a person normally spends almost 80% of his/her life in indoor environments) [83]. MOF-based sensors show advantages for environmental analyses especially in terms of high selectivity, real-time analysis, and fast response. MOF sensing materials have been developed primarily with the goal of detecting environmental contaminants in: (i) Water, such as cations, including radioactive cations, Hg^2+^, Pb^2+^, Cd^2+^, and Cu^2+^ [84], anions (PO_4_^−^, NO_2_^−^, etc.) [85], or amines [86]; (ii) air, which analysis requires MOF sensing materials with high selectivity since many gaseous chemicals have very similar molecular dimensions, for example, in the case of acetone [87], SO_2_ [88], or NH_3_ (and derivates) [89].

One recent example in this field showing the potential of MOFs for VOCs sensing consisted of a MOF film sensor array (electronic nose), which allowed the detection and isomer discrimination of VOCs in mixtures [90] The sensor was composed of six quartz crystal microbalances (QCMs) coated with different MOFs, HKUST-1, Cu(BDC), and Cu(BPDC), UiO-66, UiO-67, and UiO-68-NH_2_ (Fig. 4C). The sensor array was firstly tested in the atmosphere of pure xylene isomers, and it was noted that each QCM showed different sensitivity to the different isomers, due to unique adsorption properties. It was demonstrated, and supported by molecular simulations studies, that Cu(BDC) exhibited high affinity for *p*-xylene at low pressures and o-xylene at high pressures, while UiO-66 exhibited affinity for *o*-xylene. This was due to the rigid structure of the crystalline frameworks, which were strictly controlling the access to the adsorption site by steric hindrance and allowed thus the clear distinction of the isomers (something truly unrealizable in poor/non-crystalline materials). The performance of the sensor was analyzed with a machine learning algorithm, showing that at 100 ppm the compositions of 16 ternary mixtures were determined with an average classification accuracy of 96.5%.

## Challenges and perspectives

Tremendous progress has been made from the pioneering MOF-based sensors that first appeared in the early 2000s, such as cyano-bridged Co(II) group–based colorimetric sensors for vapor detection of diethyl ether [91], or a magnetic Cu-MOF (MOROF-1) for the detection of some solvents (methanol and ethanol) in a reversible manner [92], to more recent MOF-based sensors capable of achieving ppb detection levels, as is the case of a Zr-MOF (CJLU-1) with a fast response and very sensitive towards trinitrophenol (LOD = 83 ppb) [93].Importantly, some MOF-based sensors have been patented, which clearly demonstrates the industrial interest in the field (such as US2020269225A1, EP2520929A1, CN113624752A, US9983124B2, US9546887B1). However, there is still plenty of room for improvement.

Current challenges to be faced are related to optimizing the intrinsic sensing properties of MOFs (i.e., such as selectivity, sensitivity, responsive properties, long-term stability, reusability) by means of careful and rational MOF design approaches, as well as by developing innovative or improved synthetic strategies to precisely control the ultimate structure-properties-functions of MOFs. Moreover, large-scale productions of MOF-based sensors are still infeasible with current technologies, and key requirements such as ease of synthesis, reproducibility, and low production costs must be met before possible scaling, translation to industry, and device integration. In addition, environmental aspects must also be seriously considered, which implies proper environmental impact assessment, use of sustainable sources, etc., as well as health concerns, since biocompatibility and non-toxicity must be guaranteed in medical sensing applications.

### Optimization of MOF design and synthesis

More precise optimization of the MOF-based material in terms of size, morphology, porosity, surface properties, and incorporation of additional functionalities should be made in order to finely control and potentially tune the thermodynamics and the kinetics aspects of the interactions between MOFs and analytes, which will undoubtedly allow us to improve the critical sensing features of the material, such as selectivity, sensitivity, stability, response time, and reusability. A critical issue is achieving highly selective recognition, which remains unrealized for most analytes. Careful MOF design can play a critical role, as demonstrated in some examples by preparing a chiral framework that recognizes only one enantiomer in a mixture. For example, a recent patent reported the controlled synthesis of enantiopure MOF structures for the detection of chiral odorants such as R- and S-limonene (EP3964830A1).

Currently, the most employed techniques for the synthesis of MOFs are the diffusion method and the solvothermal method [94]. On the one hand, the diffusion method, based on the gradual convey of reactants in solvent (or gel), requires the absence of interfering species (that can affect the growth of the crystals), and the strict control of the diffusion parameters (temperature, humidity, etc.). On the other hand, the solvothermal method can produce crystals with high porosity, purity, and surface area. This is the case of a patent describing a nitrogen-rich Co-MOF used to prepare a highly sensitive fluorescent sensor (CN114213671A). However, solvothermal methods generally require high-temperature, high-boiling polar solvents (e.g., dimethylformamide), and are limited by the use of soluble precursors, which reduces the possible structures of MOFs. Alternative non-conventional methods for the synthesis of MOFs allow overcoming some of the drawbacks of conventional routes. For example, the electrochemical method can produce MOFs under mild reaction conditions. Ultrasonic-assisted and microwave-assisted methods [95–97] can produce uniformly seeded MOFs with better size control [98, 99]. This is the case of a recent study for the preparation of Co-MOFs (TMU-51) by an ultrasound-assisted technique, in which, by tuning the power of ultrasound, smaller particles with controlled morphology were achieved, which finally resulted in an improved sensing performance for nitrophenol detection [100]. Therefore, advances in unconventional synthetic methods are expected to lead to significant advances in MOF synthesis in the coming years.

Furthermore, the limited long-term stability of many MOFs under working conditions, especially under humid, acidic, or alkaline conditions, at high temperature or pressure, is an important drawback, which undoubtedly will affect the sensing performance of MOF-based sensors and eventually shorten their lifetime. To overcome this problem, besides the selection of MOFs with highly robust coordination bonds connected by high valent metal centers and multidentate hydrophobic ligands, new surface functionalization strategies to improve their stability, for example, through polymeric surface coatings, should be explored. It is worth noting that the stability needs to be evaluated under real working conditions, that is, using real samples such as dirty environmental samples or complex human body fluids, to truly determine the feasibility and reliability of the prepared MOF-based sensor in real scenarios.

Nevertheless, further systematic investigations on correlating experimental synthetic conditions, structural MOF features, adsorption enthalpies, kinetics, and sensing performance must be considered. This will greatly contribute to fully understanding the interactions and sensing mechanisms involved in reported MOF-based sensors, and consequently reoptimizing the MOF structure to reach the desired sensing performance. In this context, exhaustive and reliable characterization tools are fundamental. These techniques are aimed at studying the morphology, the chemical/structural composition, the particle number concentration, and the functionalization with molecules/inorganic particles, and the stability of the MOFs. Currently, most used techniques include electron microscopy, both scanning (SEM) and transmission (TEM), for determining the morphology (size and shape) and usually coupled to energy dispersive X-ray (EDX) detector for elemental mapping analysis; dynamic light scattering (DLS), Z-potential measurements, and nanoparticle particle tracking analysis (NTA) to study the motional behavior of MOFs in solution, including hydrodynamic size, surface charge, colloidal stability over time, and absolute concentrations (number of particles/mL); spectroscopy techniques such as ultraviolet–visible (UV–vis) and photoluminescence for determining optical properties; X-ray diffraction (XRD) to assess the crystallinity and structure; X-ray photoelectron (XPS) to determine the elemental composition and oxidation states of elements; thermogravimetric analysis (TGA) to evaluate the thermal behavior; N_2_/CO_2_/CH_4_ adsorption isotherms to study the porosity; inductively coupled plasma mass spectrometry (ICP-MS) for elemental analysis; nuclear magnetic resonance (NMR) for characterization of the linkers and surface functionalization; and chromatographic techniques (e.g., HPLC–DAD) for determining functionalization efficiency. Other more sophisticated but less accessible techniques include, for example, high-resolution TEM (HRTEM), cryogenic TEM (cryo-TEM), or annular dark-field imaging in scanning transmission electron microscopy (HAADF-STEM) for atomic-scale resolution. Recent trends in characterization techniques target, among others, the in situ analysis of MOF crystal growth [101], and the fine study of the spatial proximity between linkers, metal clusters, and the eventual guest molecules/particles [102].

### Computational studies

A powerful strategy still poorly explored for the synthesis of MOF-based material for sensing applications is the use of computational techniques. In reality, it is difficult to combine all the MOF design aspects required for an “ideal sensor” if experimental research is not aided by computation and theoretical chemists. In the process of designing a MOF for sensing, the ideal approach is to first define the sensing application (specific analyte, working conditions since this will imposes stability requirements for the MOF, etc.) to subsequently (and by taking into consideration available linkers and suitable metals) to perform computational calculations ought to give trial MOFs structure, as digital ideal prototypes [103]. Once selected and prepared the MOF, computational tools are also highly useful to understand the mechanism of interactions in order to optimize the synthetic parameters [104, 105]. For example, it was possible to evaluate the best sensing performances among nine different MOFs for the detection of gases by a Kullback-Liebler divergence study [106]. In other works, computational screening investigations of a comparatively limited library of MOFs were able to identify MOFs capable of discriminating between relatively similar molecules (e.g., xylenes and TNT) [107], or to identify one MOF with a strong preference for sorption of xenon versus krypton, in both cases by means of grand canonical Monte Carlo (GCMC) simulations [108]. Computational studies were also carried out by means of density functional theory calculation to evaluate the potential of several MOFs to detect biomarkers of SARS-CoV-2 [109]. Despite progress in this direction, the implementation of high throughput screening of MOFs for highly specific analyte adsorption/recognition will only be possible when computational techniques become routinely available, and more reliable computational methods for a wider scope of molecule/MOF interactions are also required.

### Signal transduction

Most of the MOF-based sensors reported to date rely on optical or electrochemical transduction mechanism. On the one hand, a better understanding of the function of the MOFs in each sensing scheme, not only in the recognition-sorption process but especially in how this analyte-framework interaction is transduced into a detectable signal, is crucial to fully exploit the potential of MOFs and to design more advanced transduction architectures. Very few works make a clear explanation of the working transduction mechanism and compare it with other reported mechanism. On the other hand, taking advantage of the multifunctional potential of MOFs, MOF composites integrating either NPs (e.g., plasmonic, magnetic, fluorescent), enzymes, or responsive polymers, should be more explored to endow the MOF with additional responsiveness, and providing thus more opportunities in the sensor design.

### Scale-up production

Nowadays, only a small number of companies sell MOFs, with a few crystalline structures commercially available out of the thousands reported. Chemical companies such as BASF and MOF Technologies have been improving manufacturing MOF techniques, achieving the full development of industrial scale-up processes for some remarkable MOF compounds. Indeed, BASF company claims to be the pioneer in the large-scale production of MOFs by developing an electrochemical method for the industrial preparation of HKUST-1 (US8163949B2 patent). BASF achieved a remarkable breakthrough when the hydrothermal synthesis (at the tonne level) of Basolite A520 was fully optimized. BASF currently has a portfolio of various MOFs sold under the trade name Basolite™.

The MOF type and selection of the raw materials greatly impact the large-scale implementation because the price must be as low as possible. In this sense, oxides and sulfates are preferentially chosen as metals centers, and carboxylic acids (e.g., terephthalic, isophthalic, and formic acids) are selected as the basis of the organic linkers instead of those more complex and not readily available or highly expensive [110]. Moreover, the multi-Kg production of MOFs is determined by the synthetic method in terms of easiness, temperature, and pressure conditions, use of acids and bases, batch-to-batch reproducibility, processability, and production costs. Regarding the simplicity and reproducibility of the synthesis, the diffusion and solvothermal methods are generally slow and difficult to apply on a large scale, especially because of the use of strong bases and acids, high temperature, and the difficulty of kinetically controlling the growth of MOF crystals in large reactors. On the other hand, electrochemical methods are sensitively influenced by small variations in current density, which significantly affects purity and yields. Mechanochemical synthesis can cause MOF crystals to amorphize, while microwaves and ultrasonics consume too much energy, and irradiation can destroy the MOF structure. The cost and potential scalability of the purification steps required after MOF synthesis must also be considered, as the filtration, washing, and drying procedures are often time-consuming and expensive, given the amount of solvent required and the length of time required (including waste disposal).

Despite the mentioned drawbacks, non-conventional technologies are showing promising results in the large-scale production of MOFs, as is the case of the multi-Kg (*ca.* 771.6 kg/m^3^/day) microwave-assisted continuous-flow synthesis of MIL-100(Fe) [111] or the multi-grams sonochemical synthesis of amine-functionalized metal–organic framework/graphene oxide nanocomposite [112]. Some patents have also explored new synthetic paths for the production of MOFs, such as in the case of a novel synergistic microwave-ultrasound-assisted technique (CN112452357A), or the synthesis of MOFs by flow-chemistry methods (US2016346757A1). Thus, engineering approaches may provide solutions to achieve a simple and highly reproducible synthesis of MOFs to prepare sensors in large scale. The processability of MOFs depends instead on the physical nature of the MOFs and the possibility of integrating them into the measurement system or device during the production process or in a later step. Most MOFs are currently produced as a powder product, which has significant handling, toxicity, and processability issues. Therefore, there are many research efforts focused on the production of MOFs in dispersed forms, including gels, membranes, or films [113], as in the case of a patented Zn, Fe, Cu, or Cd-MOF films used to improve the performance of a chloroform gas QCM sensor (CN109799159A), or a patented fluorescent ink based on MOFs (CN114350207A).

### Environmental aspects

The need for sustainable development is crucial in all aspects of life, including industrial production as well. However, the actual shift from a petroleum-based, energy-inefficient, and environmentally unfriendly industry to a sustainable industry is still far from taking significant steps forward. What seems most likely is a “green washing” of old methods (i.e., the appearance of sustainable production with no metrics or tests to prove the fact), also involving R&D in MOF production and all the manufacture of MOF sensors. In fact, most research articles related to MOFs do not explore the topic of sustainable development (beyond some mere theoretical considerations), nor do they calculate some simple green metrics, such as the E-Factor (the amount of waste produced in a process *per* kg of product [114, 115]). Some good works have started to investigate environmental aspects, such as the use of sustainable resources (water instead of more toxic organic solvents, earth-abundant transition metals instead of expensive precious metals, use of organic linkers derived from biomass, etc.), carrying out reactions under mild conditions, thus limiting energy consumption, reducing the amount of waste produced, using safer chemical products, etc. These works have certainly highlighted the issue of sustainable development in the field as well but, considering the mature level of MOFs, a substantial step forward should be taken. In principle, for a correct analysis, life cycle analysis (LCA) and life cycle cost (LCC) should be considered as fundamental as any of the typical characterization techniques performed. However, considering the complexity of these analyses, at least the reported sustainable characteristics of a MOF or a MOF-based sensor should be corroborated with objective quantitative data, such as green metrics.

### Health aspects

Biosafety is a major concern when developing materials for biomedical applications. Therefore, more systematic in vitro and in vivo studies for investigating the toxicity of MOF-based sensors are mandatory in these cases before their translation to clinical evaluation. Very few studies on the in vivo toxicity of MOFs have been conducted to date [116] clearly limiting the progress of MOF-based biosensors to some extent. This issue applies only to the case of sensors incorporated in the body for in situ measurements, such as the case of wearable biosensors placed directly on the human skin or other part of the body for in situ and/or real-time measurements of analytes in body fluids or tissues; however, it does not apply to sensing methods performed in the laboratory on biological samples (blood, plasma, urine, etc.). Due to this safety requirement, the use of mercury as the electrode in many electrochemical sensors has been replaced by more biocompatible bismuth and gold electrodes to develop wearable biosensors [117].

## Conclusions

The strength of MOFs over other materials strongly relies on their well-defined structures, having linking units which are amenable to chemical modification by following a rational “design-for-purpose” approach. This allows establishing structure–property-function relationships, which in turn will allow access to the desired sensing properties in terms of selectivity, sensitivity, responsiveness, stability, etc.

Due to continuous research efforts directed at the synthesis and optimization of new materials, MOF-based sensors are on the way to improving their performance, and some are expected to reach commercialization in the next few years. In fact, the increasing number of patents for new MOFs with sensing properties and sensing devices integrating MOFs clearly supports the bright future of MOF sensors in diverse industrial applications. The open issues and challenges discussed above point to future directions in the progress of MOF-based sensors. By solving these problems, the researchers will take important steps to pave the way towards the ultimate goal of having MOF-based sensors on the market.

Finally, it should be noted that the development of MOF-based sensors from the structural design to the fabrication of the analytical sensing device is a multidisciplinary task, and therefore the involvement of synthetic chemists, material science scientists, theoretical chemists, engineers, and clinicians (in case of biomedical sensors) is necessary to ultimately succeed in translating academic research into industry and/or real-world applications.
